# Pyrone-Based Inhibitors of Metalloproteinase Types 2 and 3 May Work as Conformation-Selective Inhibitors

**DOI:** 10.1111/j.1747-0285.2011.01148.x

**Published:** 2011-08

**Authors:** Jacob D Durrant, César A F de Oliveira, J Andrew McCammon

**Affiliations:** 1 Howard Hughes Medical Institute, Center for Theoretical Biological Physics, Department of Chemistry & Biochemistry, University of California at San DiegoLa Jolla, CA 92093-0365, USA; 2 Biomedical Sciences Program, University of California at San DiegoLa Jolla, CA 92093-0365, USA; 3 Department of Pharmacology, University of California at San DiegoLa Jolla, CA 92093-0365, USA

**Keywords:** computer-aided drug design, docking, inhibitors, matrix metalloproteinases, protein flexibility

## Abstract

Matrix metalloproteinases are zinc-containing enzymes capable of degrading all components of the extracellular matrix. Owing to their role in human disease, matrix metalloproteinase have been the subject of extensive study. A bioinorganic approach was recently used to identify novel inhibitors based on a maltol zinc-binding group, but accompanying molecular-docking studies failed to explain why one of these inhibitors, AM-6, had approximately 2500-fold selectivity for MMP-3 over MMP-2. A number of studies have suggested that the matrix-metalloproteinase active site is highly flexible, leading some to speculate that differences in active-site flexibility may explain inhibitor selectivity. To extend the bioinorganic approach in a way that accounts for MMP-2 and MMP-3 dynamics, we here investigate the predicted binding modes and energies of AM-6 docked into multiple structures extracted from matrix-metalloproteinase molecular dynamics simulations. Our findings suggest that accounting for protein dynamics is essential for the accurate prediction of binding affinity and selectivity. Additionally, AM-6 and other similar inhibitors likely select for and stabilize only a subpopulation of all matrix-metalloproteinase conformations sampled by the *apo* protein. Consequently, when attempting to predict ligand affinity and selectivity using an ensemble of protein structures, it may be wise to disregard protein conformations that cannot accommodate the ligand.

Matrix metalloproteinases (MMPs) are zinc-containing enzymes capable of degrading all components of the extracellular matrix. They are generally grouped into four classes depending on the matrix component degraded: collagenases (MMP-1, MMP-8, MMP-13), gelatinases (MMP-2, MMP-9), stromelysins (MMP-3, MMP-10, MMP-11), and membrane-type MMPs (MT-MMPs) (1). Owing to their role in vascular disease (2), asthma (3–5), arthritis, multiple sclerosis, Alzheimer’s disease (6), and cancer (7–9), MMPs have been the subject of extensive study. To date, several potent inhibitors have been identified, including hydroxamates (10); thiols (11); carbamoylphosphonates (12,13); hydroxyureas (14,15); hydrazines (16); β-lactams and squaric acids (17,18); and nitrogenous ligands (19,20).

Despite the design of these potent inhibitors, thus far only one MMP inhibitor has been approved by the FDA: periostat (doxycycline hyclate), used for the treatment of periodontitis. Preclinical studies of other MMP inhibitors have demonstrated severe side effects caused by inadequate selectivity. Rather than targeting only the MMP involved in pathogenesis, these compounds generally inhibit multiple MMPs, some of which are actually protective, as well as other metalloproteinases unrelated to pathology, e.g., ADAMs/adamalysins (21).

Motivated by the urgent need for selective MMP inhibitors, Puerta *et al.* recently used a bioinorganic approach to identify novel inhibitors based on a maltol (3-hydroxy-2-methyl-4-pyrone) zinc-binding group (ZBG). Rather than directly studying compound binding to an enzymatic active site, these potential ZBGs were screened against [(Tp^Ph,Me^)ZnOH] (Tp^Ph,Me^ = hydrotris(3,5-phenylmethylpyrazolyl)borate), a bioinorganic molecular model that mimics the MMP active site (22) but is more amenable to mechanistic, structural, and spectroscopic studies (23–27). Subsequent molecular modeling of the enzyme active site revealed that combining this ZBG with an amide linker permits easy access to a hydrophobic, druggable binding pocket (S1′) (10,11,28) adjacent to the active-site zinc cation. A computer fragment-docking program was used to predict the MMP-2 and MMP-3 binding affinity of several composite compounds formed by adding small-molecule fragments to the maltol ZBG (29). X-ray crystallographic data were used to build the receptor models, and fragments were selected based on the earlier work of Hajduk *et al.* (30). Experimental study revealed that three of the composite compounds, those formed by adding biphenyl (**AM-2**), biphenyl cyanide (**AM-5**), and triphenyl (**AM-6**) fragments to the ZBG, respectively, were selective for MMP-3 over MMP-2 (Table 1). Although accompanying fragment-docking calculations confirmed **AM-2** and **AM-5** selectivity for MMP-3 over MMP-2, these theoretical predictions failed to confirm the approximately 2500-fold selectivity of fragment **AM-6** for MMP-3.

**Table 1:** Experimentally measured IC_50_ values (μm) for the inhibitors AM-2, AM-5, and **AM-6** against MMP-2 and MMP-3^a^

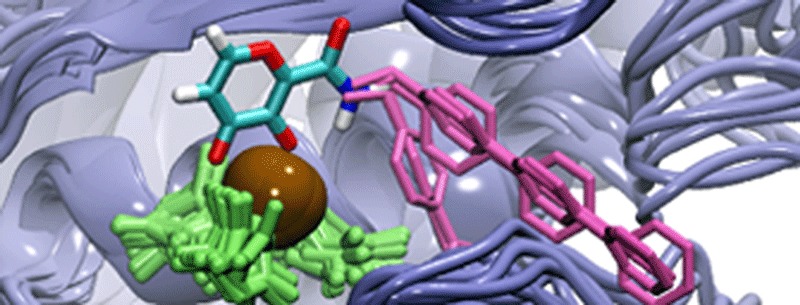

A number of studies have suggested that the MMP active site is highly flexible, leading some to speculate that differences in active-site flexibility among the different MMPs could explain specificity. In their previous work, Yuan *et al.* (31) studied the backbone amide dynamics of the MMP-3 catalytic domain using ^15^N NMR relaxation measurements. Hydroxamate- and thiadiazole-containing ligands, which bind to the S1′–S3′ (right side) and S1–S3 (left side) regions of the active site (Figure S1), respectively, were used to identify inhibitor-specific changes in the molecular dynamics (MD) of the catalytic domain. Yuan *et al.* also observed that the S1–S3 binding pockets were relatively rigid, while the S1′–S3′ pockets were highly flexible.

In another study, de Oliveira *et al.* carried out MD simulations to evaluate the dynamics of MMP-2 and MMP-3 free in solution. The authors confirmed that the S1′–S3′ pockets are highly mobile in both systems while further demonstrating that the MMP-2 and MMP-3 S1′ binding pockets nevertheless have markedly different dynamics. Specifically, MMP-3 tends to sample states in which the hydrophobic, tunnel-like S1′ pocket is often fully open, while MMP-2 tends to sample states in which the S1′ pocket is closed or at the most only partially open. By directly measuring the S1′ pocket volumes of MMP structures extracted from MD simulations, Durrant *et al.* (32) further confirmed that MMP-3 tends to be either fully open or closed, while MMP-2 is more apt to adopt intermediate states.

These studies suggest that accounting for protein flexibility may be critical for the accurate prediction of small-molecule binding affinities *in silico*. To extend the bioinorganic approach in a way that properly accounts for MMP-2 and MMP-3 dynamics, we here investigate the predicted binding modes and affinities of the ZBG-**AM-2**, ZBG-**AM-5**, and ZBG-**AM-6** compounds docked into multiple structures extracted from MMP-2 and MMP-3 MD simulations. Our findings suggest that these inhibitors select only those MMP conformations that are amenable to S1′ binding. In trying to predict selectivity, protein conformations that cannot accommodate the ligand should be disregarded.

For MMP-2, the S1′ pocket adopts a continuous spectrum of conformations from closed to open. The occasional fully open conformations, as well as many of the intermediate conformations, have S1′ pockets that can accommodate a bound ligand. MMP-3 dynamics, on the other hand, are more binary, with S1′ pockets that are generally either fully open or closed. It is only when the closed MMP-3 conformations are disregarded that the selectivity of all three inhibitors can be correctly predicted, suggesting that the compounds may in fact select for and stabilize accommodating protein conformations *in vitro*.

## Methods and Materials

### Molecular Dynamics (MD) simulations

The crystal structures of human matrix metalloproteinase-2 (MMP-2, PDB ID: 1QIB) and matrix metalloproteinase-3 (MMP-3, PDB ID: 1G4K) were used to build the initial models. Basic residues like arginine and lysine were protonated, and acidic residues like aspartate and glutamate were deprotonated. The histidine residues were assumed to be neutral at physiological pH. Except for the histidine residue located in the structural domain (His 179), which was protonated in the epsilon position, all histidine residues were protonated in the delta position. To maintain the correct orientation of the structural and catalytic zinc and histidine residues, the distances and bending angles defined by the zinc and the coordinating histidine nitrogen atoms were maintained by applying harmonic restraints. The partial charges of the imidazole rings of the histidine residues and the zinc atom were calculated using the RESP program (Figure S2, Table S1). The molecular electrostatic potential was calculated at the HF/6-31G* level. All other protein residues were assigned partial charges according to the AMBER-99SB force field (33).

Following the initial preparation, each system was subjected to 500 steps of steepest-descent and 1000 steps of conjugate-gradient minimization. Each of the minimized structures was then solvated in cubic boxes of pre-equilibrated TIP3P water molecules (34) extending 10 Å beyond the protein atoms themselves in all three dimensions. Sodium cations were next added to each system as needed to ensure electrical neutrality. The systems were again minimized for 500 steps of steepest-descent followed by 2000 steps of conjugate-gradient minimization using constant-volume periodic boundaries, with the protein and counterion atoms fixed, to relax the waters.

To achieve the correct system density, each system was next subjected to 50 ps of MD simulation with an NPT ensemble (*T* = 298 K, *P* = 1 bar) in which only the water molecules were allowed to move. Next, the systems were again energy minimized for 500 steps of steepest descent and 1000 steps of conjugate-gradient minimization. To heat each system, a 500-ps MD simulation using the NVT ensemble (*T* = 298 K) was performed, where the temperature varied gradually from 0 to 300 K. The systems were further relaxed by applying 40 ns of MD simulation using the NVT ensemble at constant temperature (*T* = 298 K). During the NVT simulations, all atoms were allowed to move freely, except for those subject to the aforementioned internal restraints, as well as those subject to SHAKE constraints placed on bonds to hydrogen atoms (35). All minimizations and MD simulations were carried out using the AMBER MD computer package (36).

### Positioning the ZBG and fragment docking

**Figure 1:**
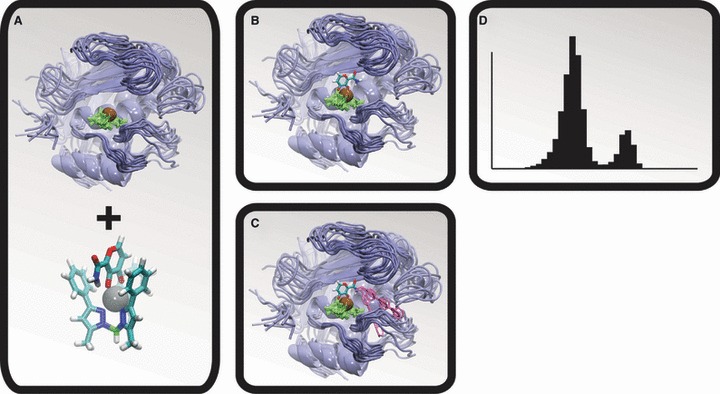
The molecular dynamics (MD)/docking protocol. The matrix metalloproteinase (MMP) protein is shown in blue, the active-site zinc cation is shown in brown, the zinc-coordinating histidine residues are shown in green, and representative LUDI-docked fragments are shown in pink. (A) A MD simulation was used to generate numerous MMP conformations. (B) A model of the zinc-binding group was positioned relative to the protein configurations by aligning a crystal structure of the bioinorganic model to the MMP active sites. (C) LUDI was used to dock molecular fragments into the S1′ active sites. (D) The resulting spectrums of LUDI scores were subsequently used to calculate ensemble-average scores.

Five thousand frames were extracted at regularly spaced intervals from both the MMP-2 and the MMP-3 simulations. These 5000 frames were aligned by the atoms of their active-site zinc cations and the three coordinating histidine residues (Figure 1A). A crystal structure of maltol bound to [(Tp^Ph,Me^)ZnOH], a bioinorganic model of the MMP active site, was then used to position the maltol ZBG correctly relative to the aligned frames (Figure 1B).

**Figure 2:**
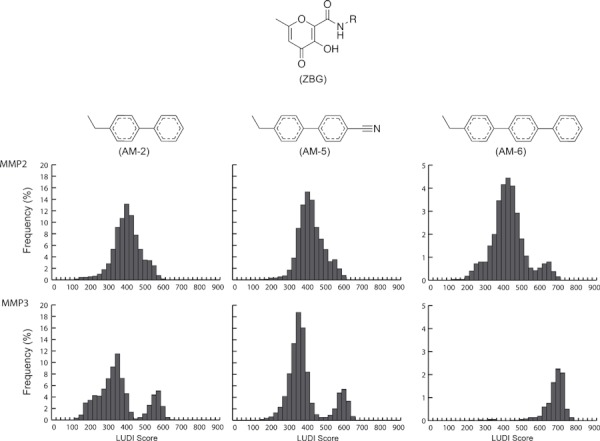
LUDI-score histograms. Fragments attached to the zinc-binding group *via* an amide linker (*R* = AM-2, AM-5, or AM-6) were docked into frames extracted from *apo* molecular dynamics simulations of MMP-2 and MMP-3. The LUDI scores were binned into histograms according to the docked fragment and receptor target. Note that a different scale has been used for AM-6 to facilitate visualization.

For each of the 5000 frames of both simulations, the docking program LUDI [Accelrys (37–39)] was used to dock three small-molecule fragments (AM-2, AM-5, and AM-6) into the active-site-positioned maltol ZBG (Figures 1C and 2), producing a spectrum of LUDI scores from which ensemble averages were ultimately calculated (Figure 1D). The ZBG amine hydrogen atom that pointed toward the S1′ pocket was selected as the link site. The following LUDI parameters were used: maximum alignment angle 20°, maximum alignment root-mean-square deviation (RMSD) 0.6 Å, search radius 11 Å, rotate bonds two at a time, preselect 4.0, minimum separation 3.0, lipophilic density 40, polar density 40, minimum surface 0, link weight 1.0, lipophilic weight 1.0, H-bond weight 1.0, aliphatic aromatic off, reject bifurcated off, no unpaired polar off, electrostatic check off, minimum score 0, maximum fits 8000, maximum hits all, maximum unfilled cavity 0, energy estimate 1 scoring function, and best fit.

### Determining the binding mode

**Figure 3:**
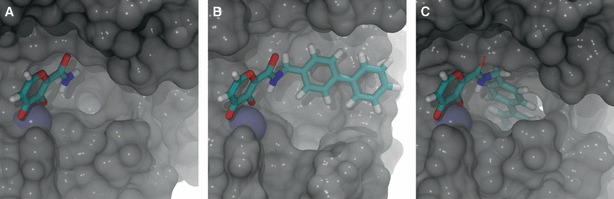
Three distinct conformations of the matrix-metalloproteinase active site, extracted from an molecular dynamics simulation. Some protein residues have been removed to facilitate visualization. The protein is shown in gray, the active-site zinc cation is shown in ice blue, and the ligand is colored by element. (A) Some protein conformations were not amenable to fragment addition. (B) Other conformations were amenable to fragment addition but did not allow deep binding to the S1′ subsite. (C) Others were amenable to both fragment addition and deep binding.

Analysis of the LUDI docking results revealed two possible binding modes: one in which the added fragment fit deeply into the S1′ pocket (Figure 3C) and another in which S1′ was not amenable to deep binding (Figure 3B). To distinguish between these two binding modes, for each frame we calculated the distance between a key S1′ carbonyl oxygen atom and the nearest atom of the LUDI-docked fragment. The carbonyl oxygen atoms of leucine 115 and leucine 111 were used for MMP-3 and MMP-2, respectively. If this distance was <5 Å, the fragment was thought to bind deep in the S1′ pocket. Otherwise, binding was considered to be superficial.

## Results and Discussion

The purpose of this study is to extend the bioinorganic approach originally pioneered by Puerta *et al.* (29) in a way that properly accounts for MMP-2 and MMP-3 dynamics. To understand the molecular basis of the observed MMP-3 selectivity of ZBG + amide linker + AM-6 (Figure 2), we explored the role protein flexibility might play in inhibitor binding for this system.

### The importance of protein flexibility

Numerous studies have demonstrated the important role of protein flexibility in ligand binding (40–45). For example, a recent MD simulation of HIV integrase revealed a previously uncharacterized binding trench that was not apparent from inspection of the static crystal structures alone. This trench was subsequently exploited in the design of Isentress (raltegravir), an HIV drug approved by the FDA in 2007 (42). A recent MD simulation of cruzain, the principal cysteine protease of the eukaryotic parasite *Trypanosoma cruzi*, likewise revealed a potentially druggable ‘cryptic binding site’ (46).

Accounting for protein flexibility can also improve the accuracy of virtual screening. For example, in a new virtual-screening protocol called the relaxed complex scheme (40), a library of compounds is docked into multiple protein conformations extracted from an MD simulation to account for full protein flexibility. When the compounds are ranked by an ensemble-based score rather than by the score associated with docking to the static crystal structure alone, hit rates are often improved. The relaxed complex scheme has been used to identify inhibitors of FKBP (47), HIV integrase (42), *Trypanosoma brucei* RNA editing ligase 1 (44), and *T. brucei* UDP-galactose 4′-epimerase (48), among others.

As NMR, X-ray crystallographic, and MD-simulation studies have shown that MMPs have highly flexible active sites (32,49–54), accounting for protein flexibility may be especially important when trying to predict MMP-ligand binding. More specifically, MD studies have revealed a highly dynamic S1′ pocket that can adopt both open- and semi-open conformations capable of accommodating large hydrophobic fragments, as well as closed conformations not amenable to binding (Figure 3). These simulations further suggest that MMP-2 heavily samples semi-open (intermediate) and closed states, while MMP-3 tends to sample either the fully closed or the fully open state. By docking fragments only into the static crystal structures of these proteins, the LUDI protocol used by Puerta *et al.* (29) did not account for the many receptor configurations sampled by these dynamic macromolecules. This oversight could potentially explain why previous computer calculations have failed to predict the >2500-fold MMP-3 selectivity of the **AM-6** inhibitor.

### Novel fragment docking into conformations extracted from an MD simulation

Recognizing that LUDI docking into static crystal structures fails to fully explain MMP-3 selectivity, we tested the hypothesis that accounting for full protein flexibility might improve prediction by docking **AM-2**, **AM-3**, and **AM-6** into 5000 protein configurations extracted from MD simulations of MMP-2 and MMP-3, respectively. Given that a significant number of the *apo* structures sampled by the MD simulations are not able to accommodate the molecular fragments, we hypothesized that conformational selection may play an important role in MMP inhibitor binding.

Figure 2 illustrates the LUDI fragment score distributions generated by docking into the MMP-2 and MMP-3 MD trajectories. The histograms of the docking scores obtained for **AM-2**, **AM-5**, and **AM-6** hint at the differences in the dynamics of the MMP-2 and MMP-3 S1′ pockets identified previously. Clearly, these differing dynamics have a significant impact on the docking results; while the score distributions associated with MMP-2 were unimodal for all fragments, those associated with MMP-3 were bimodal for **AM-2** and **AM-5**, and unimodal for **AM-6**. Bimodal distributions suggest multiple binding modes; visual inspection of the binding poses confirmed that the higher LUDI scores were consistently associated with fragments that docked deep within the S1′ pocket. In contrast, the lower LUDI scores were associated with fragments that docked into a shallow, closed, or semi-closed S1′ pocket conformation (Figure 3B,C).

The bimodal LUDI score distributions of MMP-3 demonstrate a clear separation between the constituent distributions characterizing each binding mode. In the case of **AM-6** docked into MMP-3, the distribution is likely unimodal only because the bulky **AM-6** fragment could not dock into a shallow, closed, or semi-closed S1′ pocket at all. These findings are in good agreement with our previous MD simulations, which show that the open and closed form of the S1′ binding pocket are indeed the most populated conformational states sampled by MMP-3, with few intermediate states (32,54).

The LUDI score distributions associated with MMP-2 likewise show good agreement with previous MD results. For MMP-2, the S1′ pocket breathes continuously from a closed to an open conformation and so can generally accommodate a bound molecular fragment. The MMP-2 MD trajectory sampled a wide range of intermediate conformational states between fully open and closed. Consequently, a unimodal score distribution is observed for **AM-2** and **AM-5** docked into the MMP-2 active site. Even if the distribution associated with **AM-6** is considered to be bimodal, the mode associated with the higher LUDI score is only rarely sampled. Regardless, this higher-score state is considerably less populated in MMP-2 than in MMP-3, where it is the only mode sampled.

### Interpretation of results, agreement with experiment

Emil Fischer (55) first proposed the lock-and-key model of ligand binding in 1894. While didactically useful, this model, which suggests that a protein exists in a single conformation that is perfectly complimentary to the ligand it binds, has fallen out of favor in light of crystallographic and NMR evidence (56–58). A number of subsequent theories have been presented describing the important role protein flexibility plays in ligand binding (59). In recent years, researchers have begun to theorize that binding often occurs *via* a population-shift mechanism, commonly called conformational selection (60–63). The theory states that an *apo* protein, in constant motion as it ‘breaths’ in solution, continuously samples many different active-site conformations. Only a certain subpopulation of these conformations is amenable to ligand binding. In the presence of a ligand, this amenable subpopulation is stabilized (i.e., ‘selected’), causing the population of conformations to shift away from those that are incompatible with binding.

Drawing on the theory of conformational selection, we postulate that in the case of the MMPs only ligand binding deep within the S1′ pocket is truly representative of bound complexes *in vitro*. As MMP-3 rarely samples intermediates states (i.e., the semi-open states of the S1′ pocket), the fragments **AM-2**, **AM-5**, and **AM-6** can only bind to, or select, conformational states in which the S1′ pocket is fully open. We consequently discarded those MMP-3 conformations with closed S1′ pockets when calculating the ensemble-average LUDI scores. In contrast, to properly calculate the ensemble-average LUDI scores of MMP-2 binding, we considered all conformational states because intermediate semi-open S1′ pockets amenable to ligand binding are commonly sampled.

The calculated average LUDI scores of **AM-6** bound to MMP-2 and MMP-3 were 440.1 ± 95.6 and 697.1 ± 52.1, respectively. Because each 100 LUDI score units corresponds to an IC_50_ increase of one order of magnitude (37,64), our results are in excellent agreement with the three-order-of-magnitude difference obtained experimentally (IC_50_ values of >50 and 0.019 μm for MMP-2 and MMP-3, respectively; Table 1) (29). The same agreement with experiment is observed when a similar protocol is applied to **AM-2** and **AM-5**. The average LUDI score of **AM-2** bound to MMP-2 and MMP-3 was 413.1 ± 76.3 and 534.6 ± 94.2, respectively. The roughly 100 units difference is in good agreement with the experimentally measured IC_50_ values of 9.3 and 0.24 μm, respectively (Table 1). Similarly, the average LUDI score of **AM-5** bound to MMP-2 and MMP-3 was 431.3 ± 69.9 and 567.4 ± 84.9, respectively, again comparing well with the experimentally measured IC_50_ values (0.61 and 0.01 μm, respectively; Table 1). We note that this agreement with experiment was not obtained when the closed MMP-3 conformations were retained in the calculation, suggesting that they are not biologically relevant to binding *in vitro*.

## Conclusions

Based on this analysis, our results suggest that the ZBG with attached fragments **AM-2, AM-5**, and **AM-6** may bind to MMP-2 and MMP-3 *via* a conformational selection mechanism. The dynamics of the S1′ binding pocket reveal that the receptor samples three principal conformational states: (a) fully closed, incompatible with binding/docking (no LUDI scores, Figure 3A); (b) semi-open, compatible with weak binding (Figure 3B); and (c) fully open, compatible with strong binding (corresponding to the higher mode of the LUDI score distributions, Figure 3C).

These results confirm the findings of previous studies that suggest that accounting for protein flexibility is critical to the theoretical prediction of small-molecule binding affinities, especially when studying MMPs. Our ensemble-based docking analyses also indicate that differences in the dynamics of MMP-2 and MMP-3 likely explain the selectivity of the **AM-6** compound for MMP-3.

## Supporting Information

**Figure S1**. The various subsites of the MMPenzymatic pocket.

**Figure S2**. Residues with non-standard,RESP-calculated partial charges.

**Table S1.** The partial charges used in non-standard residues.
