# Supplementary material for: Pyrone-Based Inhibitors of Metalloproteinase Types 2 and 3 May Work as Conformation-Selective Inhibitors
Source: Chem Biol Drug Des. 2011 Aug;78(2):191–8. doi: 10.1111/j.1747-0285.2011.01148.x (PMC3135671; doi:10.1111/j.1747-0285.2011.01148.x)
Supplement: Supplementary file 1 [file cbdd0078-0191-SD1.doc]

**Supporting Information**

Pyrone-Based Inhibitors of Metalloproteinases Types 2 and 3 May Work as Conformation-Selective Inhibitors

Jacob D. Durrant, César Augusto F. de Oliveira, and J. Andrew McCammon

**Table of Contents**

Figure S1 …………………………………………………………………………….. S2

Figure S2 …………………………………………………………………………….. S3

Table S1 ……………………………………………………………………………... S4


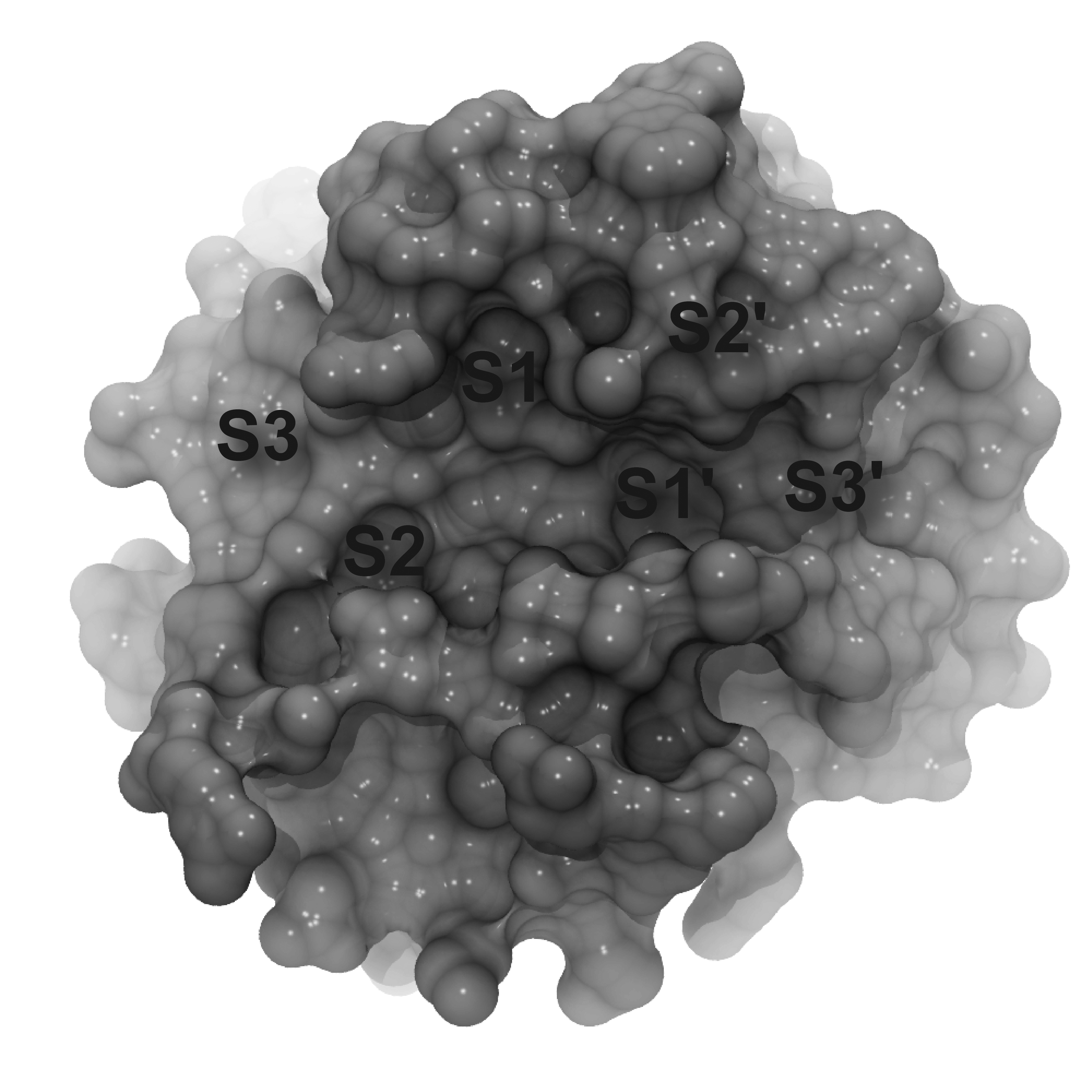


**Figure S1**. The various subsites of the MMP enzymatic pocket.


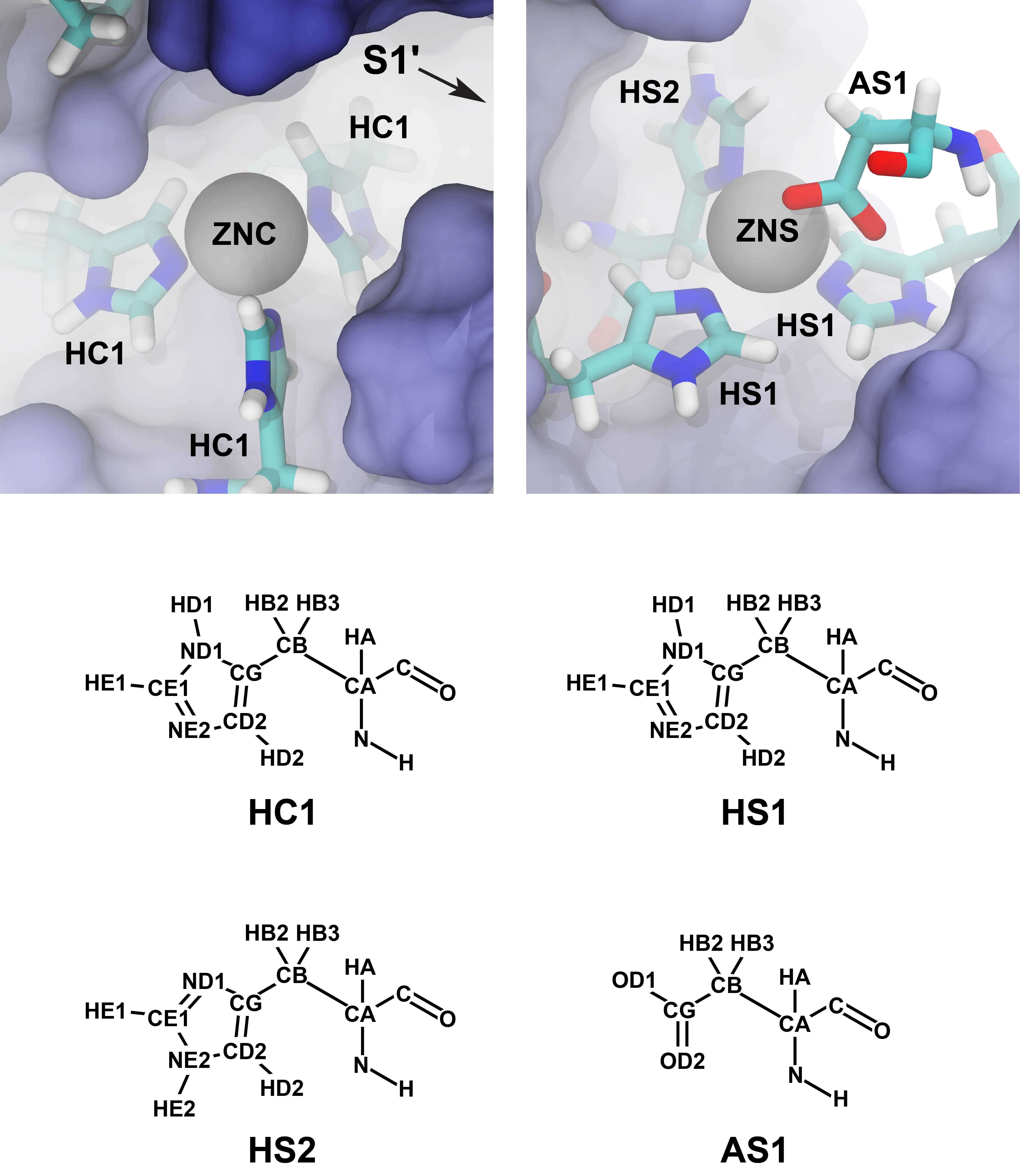


**Figure S2**. Residues with non-standard, RESP-calculated partial charges. Key residues from the catalytic site are shown in the upper-left panel, and key residues from the structural site are shown in the upper-right panel. Residue atom names are given below, and partial charges are shown in Table S1.

**Table S1.** The partial charges used in non-standard residues.

| **HC1** |  |  | **HS1** |  |
| --- | --- | --- | --- | --- |
| **Atom Name** | **Partial Charge** |  | **Atom Name** | **Partial Charge** |
| N | -0.415700 |  | N | -0.415700 |
| H | 0.271900 |  | H | 0.271900 |
| CA | 0.018800 |  | CA | 0.018800 |
| HA | 0.088100 |  | HA | 0.088100 |
| CB | 0.146076 |  | CB | 0.202135 |
| HB2 | 0.043188 |  | HB2 | -0.003978 |
| HB3 | 0.043188 |  | HB3 | -0.003978 |
| CG | -0.042333 |  | CG | -0.022850 |
| ND1 | -0.101470 |  | ND1 | -0.218294 |
| HD1 | 0.338864 |  | HD1 | 0.352246 |
| CE1 | -0.062743 |  | CE1 | -0.089392 |
| HE1 | 0.233674 |  | HE1 | 0.239459 |
| NE2 | -0.371835 |  | NE2 | -0.017032 |
| CD2 | -0.140577 |  | CD2 | -0.206105 |
| HD2 | 0.200802 |  | HD2 | 0.211805 |
| C | 0.597300 |  | C | 0.597300 |
| O | -0.567900 |  | O | -0.567900 |
|  |  |  |  |  |
| **HS2** |  |  | **AS1** |  |
| **Atom Name** | **Partial Charge** |  | **Atom Name** | **Partial Charge** |
| N | -0.415700 |  | N | -0.516300 |
| H | 0.271900 |  | H | 0.293600 |
| CA | -0.058100 |  | CA | 0.038100 |
| HA | 0.136000 |  | HA | 0.088000 |
| CB | 0.372152 |  | CB | 0.494367 |
| HB2 | -0.080594 |  | HB2 | -0.118479 |
| HB3 | -0.080594 |  | HB3 | -0.118479 |
| CG | -0.096152 |  | CG | 0.636236 |
| ND1 | -0.013382 |  | OD1 | -0.658521 |
| CE1 | 0.041065 |  | OD2 | -0.658521 |
| HE1 | 0.199150 |  | C | 0.536600 |
| NE2 | -0.287521 |  | O | -0.581900 |
| HE2 | 0.385332 |  |  |  |
| CD2 | -0.245019 |  |  |  |
| HD2 | 0.252084 |  |  |  |
| C | 0.597300 |  |  |  |
| O | -0.567900 |  |  |  |
|  |  |  |  |  |
| **ZNC** |  |  | **ZNS** |  |
| **Atom Name** | **Partial Charge** |  | **Atom Name** | **Partial Charge** |
| ZN | 1.162000 |  | ZN | 0.282200 |
